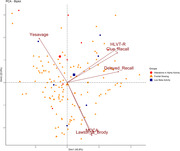# Correlation Between QEEG Patterns and Neuropsychological Profile in Patients with Cognitive Impairment from the Colombian Caribbean

**DOI:** 10.1002/alz.084782

**Published:** 2025-01-03

**Authors:** Jose Hernandez Preciado, Alex Dominguez Vargas, Wanda Torres, Yesenia Pianneta, Mauricio Medina, Marybel Sinisterra, José Vargas

**Affiliations:** ^1^ Universidad de Maimónides, Buenos Aires Argentina; ^2^ Universidad del Norte, Puerto Colombia Colombia; ^3^ Universidad Simón Bolívar, Barranquilla Colombia; ^4^ Universidad de Caldas, Manizales Colombia

## Abstract

**Background:**

Quantitative Electroencephalography (qEEG) plays a pivotal role in the assessment and categorization of cognitive impairment (CI). The integration of qEEG markers with neuropsychological test scores can predict rapid cognitive decline in neurodegenerative diseases. The aim of this study was to correlate qEEG findings with the neuropsychological profile in patients with CI from the Colombian Caribbean.

**Methods:**

A cross‐sectional study. Patients with CI (n = 151) from the northern coast of the Colombian Caribbean region were included. Cognitive function was assessed using qEEG and it was categorized as follows: low beta activity (mild CI) (n = 6), frontal slowing (moderate CI) (n = 137), and alterations in alpha activity (severe CI) (n = 8). A cuantitative and normative EEG (Z‐score) was performed using the international 10‐20 system assembly, a 36 channel EEG amplifier and normative database software‐Neurovirtual were used. Neuropsychological profiling was assessed using the following tests: Hopkins Verbal Learning Test‐Revised (HVLT‐R), Clue Recall, Delayed Recall, Memory Complaint Scale (MCS), Trail Making Test Part A and B (TMT‐A/B), Symbol‐Digit test, Lawton & Brody, Yesavage, and Montreal Cognitive Assessment (MoCA). Principal component analysis was used to assess relationships between qEEG categories and the neuropsychological profile (Figure 1).

**Results:**

Patients with alterations in alpha activity exhibited significantly lower scores on the Lawton & Brody test (p = 0.02) and MoCA (p = 0.04) and higher scores on the Yesavage test (p = 0.04). Significant positive correlations were observed between HLVT‐R and Clue recall tests (Spearman r = 0.83, p<0.001), as well as between the MOCA and Lawton & Brody tests (Spearman r = 0.74, p>0.001). In contrast, significant negative correlations were found between the Yesavage and Lawton & Brody tests (Spearman r = ‐0.45, p = 0.008).

**Conclusions:**

In this study, patients with alterations in alpha activity were correlated with lower cognitive performance and emotional impairment. This study underscores the importance of qEEG in conjunction with the neuropsychological profile in evaluating cognitive impairment, emphasizing its utility in diagnostic and therapeutic interventions in cognitive health planning.